# Dynamic Diffraction Studies on the Crystallization, Phase Transformation, and Activation Energies in Anodized Titania Nanotubes

**DOI:** 10.3390/nano8020122

**Published:** 2018-02-23

**Authors:** Hani Albetran, Victor Vega, Victor M. Prida, It-Meng Low

**Affiliations:** 1 Department of Basic Sciences, College of Education, Imam Abdulrahman Bin Faisal University, P. O. Box 2375, Dammam 31451, Saudi Arabia.; 2 Department of Physics, University of Oviedo, C/Federico Garcia Lorca No. 18, 33007 Oviedo, Asturias, Spain; vegavictor@uniovi.es (V.V.); vmpp@uniovi.es (V.M.P.); 3 Department of Physics and Astronomy, Curtin University, GPO Box U1987, Perth, WA 6845, Australia; j.low@curtin.edu.au (I.M.L.)

**Keywords:** titania, anatase, rutile, anodization, synchrotron radiation diffraction

## Abstract

The influence of calcination time on the phase transformation and crystallization kinetics of anodized titania nanotube arrays was studied using in-situ isothermal and non-isothermal synchrotron radiation diffraction from room temperature to 900 °C. Anatase first crystallized at 400 °C, while rutile crystallized at 550 °C. Isothermal heating of the anodized titania nanotubes by an increase in the calcination time at 400, 450, 500, 550, 600, and 650 °C resulted in a slight reduction in anatase abundance, but an increase in the abundance of rutile because of an anatase-to-rutile transformation. The Avrami equation was used to model the titania crystallization mechanism and the Arrhenius equation was used to estimate the activation energies of the titania phase transformation. Activation energies of 22 (10) kJ/mol for the titanium-to-anatase transformation, and 207 (17) kJ/mol for the anatase-to-rutile transformation were estimated.

## 1. Introduction

Titania is a promising and attractive raw material for its use in various applications such as hydrogen production, gas sensors, photoelectrochemical cells, and dye-sensitized solar cells because of its stability, nontoxicity, photo-durability, affordability, and excellent photocatalytic activity [1,2,3,4,5].

Anatase, rutile, and brookite are three common naturally occurring titania crystal structures [6,7,8,9]. The thermal treatment of amorphous titania at elevated temperature yields, anatase, rutile, or a mixture of these crystalline structures as the most common titania phases, whereas brookite formation is rare [10]. Titania phase transformation is influenced by several conditions, such as calcining temperature, heating rate, calcination time, synthesis method, dopants, the level of impurities, grain size, and atmospheric condition and type [11,12,13,14,15,16,17].

The effect of calcination time on the titania phase transformation has not been investigated intensively and is not yet clear and fully understood. Some studies of this effect exist in the literature, for example, titanium dioxide nanotube arrays have been heated at 500 °C/min and 10 °C/min with a calcination time of 1, 2, 4, 8, and 12 h [18]. The X-ray powder diffraction (XRD) patterns show that only the (101) anatase peak of the crystallographic plane appears after one hour of calcination, and (101) and (200) anatase crystallographic planes appear after two hours. An anatase-to-rutile phase transformation was observed after four hours of calcination, and anatase transformed completely into rutile after 12 h of calcination. This effect was studied by heating anatase in air at 950 °C for 4, 24, and 48 h [19]. Diffraction results show that an increase in calcination time increased the abundance of rutile but decreased the anatase abundance. The abundances of rutile were 5 wt % for 4 h, 65 wt % for 24 h, and 92 wt % for 48 h.

Due to their unique electronic characteristics, such as quantum-confinement effects, a high surface-area-to-volume ratio, a high mechanical strength, and a high electron mobility [3,4,20,21], one-dimensional titania nanostructures such as nanofibers, nanobelts, nanorods, nanowires, and nanotubes have attacted much interest.

Various fabrication methods have been used to synthesize one-dimensional nanostructured titania and these include the sol-gel, electrospinning, hydrothermal, and anodization methods. Electrochemical anodization is currently one of the most common inexpensive, and simple methods that is used to fabricate one-dimensional nanotube titania nanostructures [2,20,22,23]. At room temperature, electrochemical anodization of the titanium-based alloy as a cathode in an electrolyte with a platinum grid as an anode produced anodized titania nanotube arrays [2,24,25].

Previous XRD studies of as-synthesized anodized titania nanotubes showed only diffraction peaks of titanium metal phase and no peak of any titania phases (anatase, or rutile) exists, which indicates that the titania nanotubes are in the amorphous state before calcination [26,27,28,29]. Commonly, anatase phase crystallizes from amorphous titania nanotubes arrays at relatively low temperatures and subsequently transforms to rutile phase at higher temperatures. The crystalline phase transformation process of amorphous-to-anatase in the titania nanotubes arrays can be observed by using a hydrothermal treatment method under various reaction times, and at low temperature experimental conditions (~ 180 °C) [26,29].

In this study, the effect of calcination time on phase transformations, crystallization kinetics, and activation energies of anodized titania nanotube arrays was investigated using isothermal in-situ synchrotron radiation diffraction (SRD) from room temperature to 900 °C. The sample was also characterized using field emission scanning electron microscopy (FESEM), and associated energy dispersive spectroscopy (EDS).

## 2. Materials and Methods

### 2.1. Sample Preparation 

Details of the anodization technique are given in a preliminary study performed by the authors [16,30]. In summary, the anode was a titanium foil (10 × 10 × 0.1 mm^3^, 99.96% purity), the cathode was a platinum mesh, and the electrochemical anodization was carried out at constant pH = 6 electrolyte that consisted of a mixture of ammonium fluoride, ethylene glycol, and water at room temperature. The anodizing conditions were set for 20 h at a constant applied voltage of 60 V. After anodization process, the obtained samples of titania nanotube arrays were rinsed or washed with ethanol, immersed in hexamethyldililazane (HMDS), and dried in air.

### 2.2. Field Emission Scanning Electron Microscopy

Anodized titania nanotubes array surface morphologies were studied by field emission scanning electron microscope (FESEM, Zeiss, Neon, 40EsB, Oberkochen, Germany) with secondary electron at a 5-KV accelerating voltage. Samples were studied prior to and after the in-situ high-temperature SRD. Charging was prevented by platinum sputter-coating the anodized titania nanotube arrays to a 3-nm thickness. FESEM images of the material and elemental compositions of the anodized titania nanotube arrays were analyzed by energy dispersive X-ray spectroscopy (EDS) (Oxford Instruments, Abingdon, Oxfordshire, UK).

### 2.3. Measurements and Data Analysis of in-situ Isothermal Synchrotron Radiation Diffraction (SRD)

**Figure 1:**
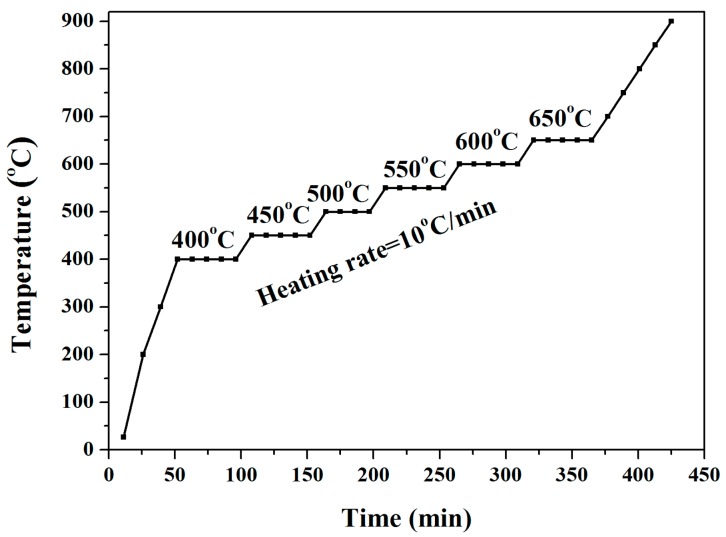
SRD heating protocol at 10 °C/min heating, 2 min/pattern acquired initially at ambient temperature, 100, 200, and 300 °C non-isothermally; 50 °C steps from 400 to 650 °C isothermally at 400, 450, 500, 550, 600, and 650 °C, and 50 °C steps from 650 to 900 °C non-isothermally.

In-situ isothermal high temperature SRD in air (powder diffraction beamline, Australian Synchrotron, Melbourne, Australia; 3° incident angle, 0.1126-nm wavelength) was used to study the high temperature crystallization kinetics of the anodized titania nanotube arrays. The specimen was mounted and heated using an Anton Parr HTK 16 hot platinum stage and an Mythen II microstrip detector (Australian Synchrotron, Melbourne, VIC, Australia). The heating protocol (10 °C/min; data acquisition of 2 min/pattern; ambient temperature, 100, 200, and 300 °C non-isothermally; 50 °C steps from 400 to 650 °C isothermally at 400, 450, 500, 550, 600, and 650 °C and 50 °C steps from 650 to 900 °C non-isothermally) for the SRD studies of the titania nanotube arrays is given in Figure 1. The residence times from 400 to 650 °C are shown in Table 1. Each pattern of the anodized titania nanotube arrays was measured over of 2θ = 5°–84°.

### 2.4. Quantitative Analysis

The Rietveld method (TOPAS software, Bruker AXS 4.2) was used for SRD patterns analysis. This accuracy of this method should be greater than that of the Spurr and Myers method as the latter uses the anatase (101) and rutile (110) peak intensities only, and the former overcomes shortcomings of the degree of crystallinity, the grain size and the preferred orientation [31,32]. The goodness-of-fit was determined from the derived Bragg *R*-factors (*R*_P_), the weighted pattern *R*-factor (*R*_wp_), and the expected *R*-factor (*R*_exp_). The relative abundances of the crystalline phase abundances of rutile, anatase, and titanium were calculated relative to temperature using an optimized sample displacement; pattern background; 2θ_0_; and peak shape, preferred orientation, lattice parameters, and scale factor for each phase. Rutile (ICSD 64987), anatase (ICSD 202242), titanium _α_ (ICSD 44872), and titanium _β_ (ICSD 76165) crystal structures were used for Rietveld refinements. Phases identification was conducted by searching the powder diffraction file database (PDF-4+2013 version: 4.13.0.6, database 4.1302) of the International Centre for Diffraction Data (ICDD) using the DIFFRAC. EVA software (2012, version 3.1).

### 2.5. Crystallization Activation Energies

Results from the isothermal phase analysis were used to estimate the activation energies for the transformations of Ti_(α + β)_-to-anatase and anatase-to-rutile.

The kinetics of isothermal crystallization of amorphous titania is described by the Avrami expression for solid-state transformations [33]:

(1) $$w_{t}=1-e^{-{\left(kt\right)}^{n}}$$

where *w_t_* is the transformed material weight fraction after time *t*. The kinetic exponent depends on the growth mechanism and titania crystal dimensionality. The Arrhenius equation gives the temperature-dependence of the reaction rate constant *k* [34]:

(2) $$k=k_{o}e^{-E/RT}$$

where *k_o_* is a constant, *E* is the activation energy (kJ/mol), *T* is the temperature (K), and *R* is the gas constant (8.3145 J/kmol). The activation energy can be determined from the gradient of the linear regression fit of the plot of ln[−ln(1 − *wt*)] versus 1/*T*. The activation energy of the titanium-to-anatase transformation was calculated from the anatase-plus-rutile weight fraction at each temperature assuming that amorphous titania crystallizes anatase, which is transformed to rutile. The activation energy of transformation of the anatase to rutile was estimated from the weight fraction for rutile at each temperature.

## 3. Results and Discussion

### 3.1. Microstructural Imaging

**Figure 2:**
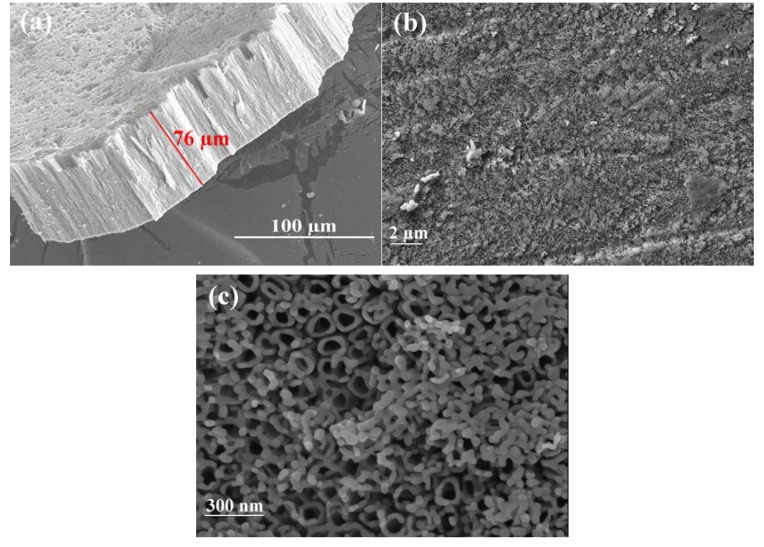
FESEM micrographs for anodized titania nanotube arrays (**a**) after the anodization process (60 V, 25 °C, 20 h), and following SRD experiments and viewed with (**b**) low, and (**c**) high magnification.

After anodizing the starting Ti substrate in an ammonium fluoride/ethylene glycol electrolyte (60 V, 25 °C, 20 h), a white homogeneous layer formed by the parallel aligned grown titania nanotubes having a mean size diameter of 60 nm and 76 microns in length, resulted on the titanium-foil (Figure 2a). Figure 2b and c present typical secondary-electron FESEM micrographs for the anodized titania nanotube arrays, following the SRD experiments with a low, and a high magnification, respectively. Some grains were distributed randomly among the titania nanotube. The grains are primarily crystalline rutile that result from the transformation of metastable anatase to stable crystalline rutile with the weight percentage of 74.74% after 900 °C thermal annealing, which takes place not only at surface level, but also in the bulk of the TiO_2_ nanotube arrays (Section Crystallization Kinetics).

**Figure 3:**
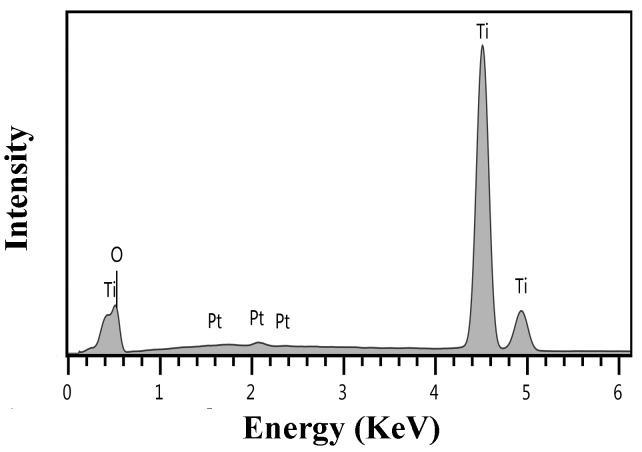
EDS spectra for heat treatment (to 900 °C) and cooling to room temperature for nanotubes of anodized titania.

The EDS spectrum of arrays of anodized titania nanotube are shown in Figure 3, with strong signatures for titanium, oxygen, and platinum, and with the platinum content resulting because of the platinum coating. Quantitative EDS analysis (conducted assuming that the platinum coating peaks are negligible, and with AZtec Software, Oxford, UK) indicates that 66.18% O K_α_ and 33.82 %Ti *K*_α_ exist, thus confirming the presence of stoichiometric titania phase [30]. The flat samples were studied by quantitative EDS, and an approximate analysis was obtained for the titania nanotubes. The nanotubes EDS was obtained from a small square to reduce problems related to the sample not being flat.

### 3.2. Crystallization Kinetics

**Figure 4:**
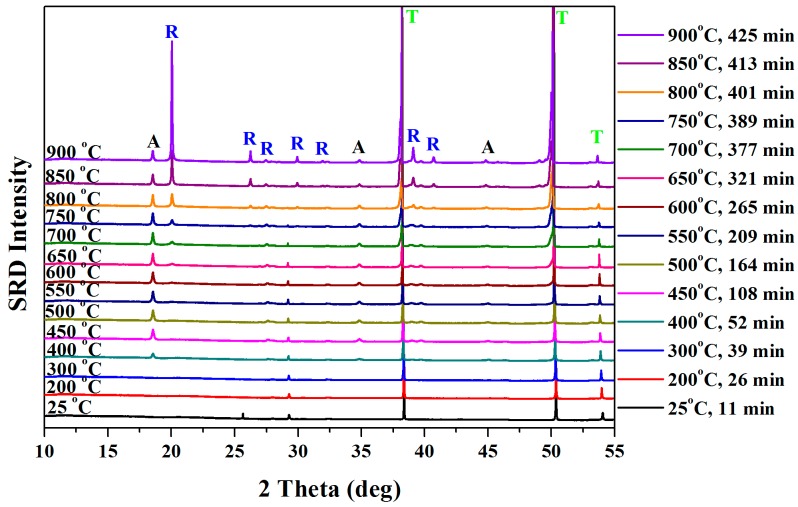
Stacked in-situ high-temperature SRD plot for as-anodized titania nanotube arrays obtained at ambient temperature; in 100 °C steps from 100 to 300 °C; and 50 °C steps from 400 to 900 °C at 10 °C/min, 2 min/pattern data-acquisition time [Ti: titanium, A: anatase, and R: rutile].

Figure 4 shows the stacked SRD plot during thermal calcination in air with an experimental SRD time, at ambient temperature; 100, 200, and 300 °C; and with 50 °C steps from 400 to 900 °C. Diffraction analysis indicates that the initial arrays of titania nanotube were amorphous, with only Ti_α_ peaks, but, at 400 °C, crystalline anatase formed. The small crystalline rutile peak indicates that the transformation of anatase to rutile commenced at 550 °C. These results are similar to the interpretation of the SRD plot for pure anodized titania nanofiber by Low and his co-workers [35]. The metastable Ti_α_ transformed into another metastable Ti_β_ at 700 °C, and both these materials transformed into titania phases at elevated temperature.

**Figure 5:**
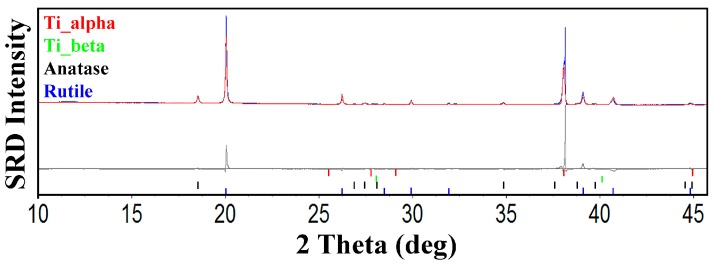
SRD Rietveld difference plots for anodized titania nanotube arrays data measured at 900 °C. Black crosses show measured patterns and solid red lines show calculated patterns. Difference between measured and calculated patterns are shown by the gray residual plot. Red, green, black, and blue bars show peak positions for Ti_α_, Ti_β_, anatase and rutile, respectively. The plots were computed with TOPAS (Version 4.2).

Relative analysis was used to calculate the relative phase levels of titanium-α, titanium-β, crystalline anatase, and crystalline rutile in the anodized nanotube arrays. The residual values for the Topas refinements *R*_wp_ and *R*_exp_ were ~ 9 and 2, respectively. The goodness-of-fit, *X*^2^, ranged from 4 to 5, which indicates that the quality of the refinements was acceptable [30,31,32,36]. Figure 5 shows typical SRD Rietveld residual plots at 900 °C with goodness-of-fit values of 4.26 for this pattern.

**Figure 6:**
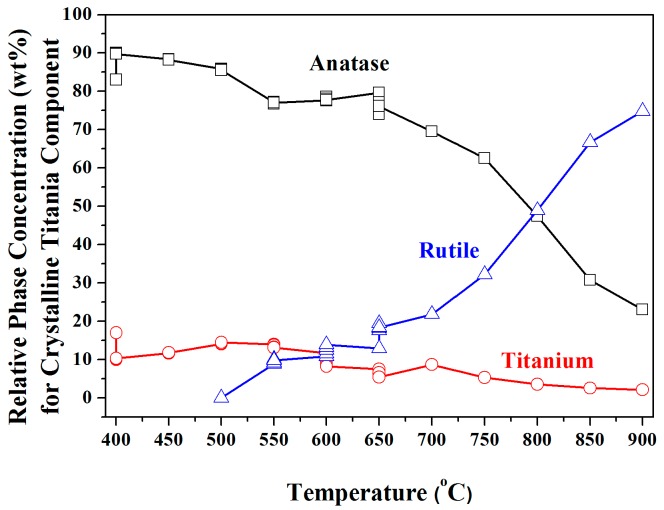
Phase abundances of titanium, crystalline anatase and crystalline rutile for anodized titania nanotube arrays calcined at 400–900 °C.

Figure 6 shows the phase abundances of Ti_(α+β)_, crystalline anatase, and crystalline rutile for the titania nanotube arrays from 400 to 900 °C. At 400 °C, and from 52 to 96 min after initiation of the experiment, the Ti_α_ transformed rapidly into anatase. Anatase grew progressively after the formation, whereas the Ti_α_ concentration was reduced steadily beyond 900 °C because of its transformation to anatase. At 550 °C, the anatase-to-rutile transformation did not commence immediately, but required 44 min for its transformation, which occurred rapidly from 600 to 650 °C. The calcination time affected the anatase-to-rutile transformation, which increased moderately with increasing calcination time at 550, 600, and 650 °C.

### 3.3. Activation Energies

**Figure 7:**
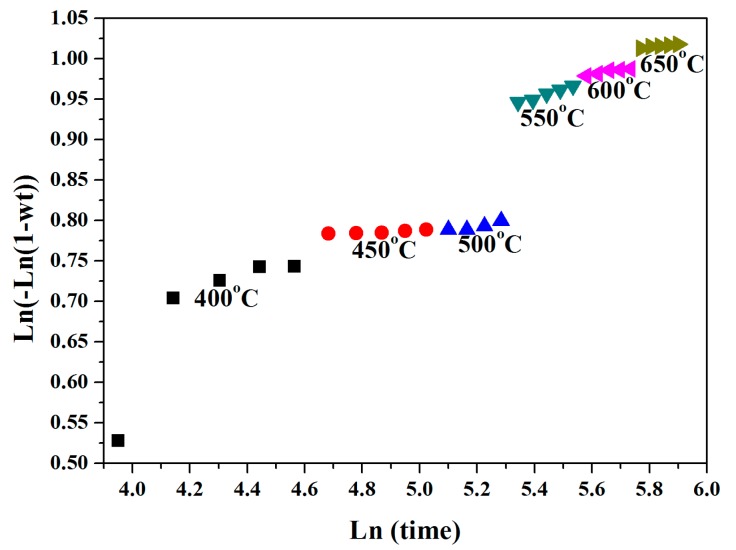
The Avrami plot used to determine the average temperature-dependent rate constant at 400, 450, 500, 550, 600, and 650 °C.

The activation energy of Ti_(α + β)_-to-anatase, and anatase-to-rutile were obtained by using the Avrami and Arrhenius equations. Figure 7 shows the Avrami plot that was used to determine the average temperature-dependent rate constant at 400, 450, 500, 550, 600, and 650 °C. Linear regressions of the Arrhenius equation were used to calculate the activation energies from the isothermal SRD data, with the assumed sequential transformations being titanium-to-anatase and then anatase-to-rutile. The corresponding activation energies for this process were 22 (10) kJ/mol for the titanium-to-anatase transformation, and 207 (17) kJ/mol for the anatase-to-rutile transformation. A comparison of the isothermal SRD anatase-to-rutile activation energies for the anodized titania nanotube arrays in this study indicate that these values are close to these of the activation energy reported in literature, such as 213 kJ/mol for powder, and membranes [37,38].

## 4. Conclusions

The effect of calcination time, crystallization kinetics, and activation energies of anodized titania nanotube arrays was investigated using in-situ high-temperature SRD from 25 to 900 °C isothermally at 400, 450, 500, 550, 600, and 650 °C. Only anodized titanium diffraction peaks were visible for calcination from room temperature to 300 °C, which indicates the formation of amorphous titania nanotubes. The titanium-to-anatase transformation started at 400 °C as seen from the crystalline anatase peaks, whereas the anatase-to-rutile transformation occurred at 550 °C as shown by the crystalline rutile peaks. Results show that an increase in calcination time increased the abundance of rutile, but the anatase abundance decreased. The activation energies from the analysis of the isothermal SRD data using the Avrami and Arrhenius equations provided estimates of 22 (10) kJ/mol for the titanium-to-anatase transformation and 207 (17) kJ/mol for the anatase-to-rutile transformation.

## Figures and Tables

**Table 1:** Heating schedule during in-situ isotermal high-temperature SRD.

Temperature (°C)	Residence time (min)
400	44
450	44
500	34
550	44
600	44
650	44

## References

[1] Gilja V., Novaković K., Travas-Sejdic J., Hrnjak-Murgić Z., Roković M.K., Zic M. (2017). Stability and synergistic effect of polyaniline/TiO_2_ photocatalysts in degradation of azo dye in wastewater. Nanomaterials.

[2] Macak J.M., Tsuchiya H., Ghicov A., Yasuda K., Hahn R., Bauer S., Schmuki P. (2007). TiO_2_ nanotubes: self-organized electrochemical formation, properties and applications. Curr. Opin. Solid. State. Mater. Sci..

[3] Xiong H., Slater M.D., Balasubramanian M., Johnson C.S., Rajh T. (2011). Amorphous TiO_2_ nanotube anode for rechargeable sodium ion batteries. J. Phys. Chem. Lett..

[4] Yanga D., Parka H., Choa S., Kima H., Choi W. (2008). TiO_2_-nanotube-based dye-sensitized solar cells fabricated by an efficient anodic oxidation for high surface area. J. Phys. Chem. Solids.

[5] Albetran H., O'Connor B., Low I. (2016). Effect of calcination on band gaps for electrospun titania nanofibers heated in air–argon mixtures. Mater. Des..

[6] Hanaor D.A., Sorrell C.C. (2011). Review of the anatase to rutile phase transformation. J. Mater. Sci..

[7] Chuangchote S., Jitputti J., Sagawa T., Yoshikawa S. (2009). Photocatalytic activity for hydrogen evolution of electrospun TiO_2_ nanofibers. ACS Appl. Mater. Interfaces.

[8] Kim D., Enomoto N., Nakagawa Z., Kawamura K. (1996). Molecular dynamic simulation in titanium dioxide polymorphs: Rutile, brookite, and anatase. J. Am. Ceram. Soc..

[9] Liu G., Wang L., Yang H.G., Cheng H.M., Lu G.Q. (2010). Titania-based photocatalysts-crystal growth, doping and heterostructuring. J. Mater. Chem..

[10] Beltran A., Gracia L., Andres J. (2006). Density functional theory study of the brookite surfaces and phase transitions between natural titania polymorphs. J. Phys. Chem. B.

[11] Ghicov A., Tsuchiya H., Macak J.M., Schmuki P. (2006). Annealing effects on the photoresponse of TiO_2_ nanotubes. Phy. Status. Solidi. A.

[12] Liu Z., Yan X., Chu W., Li D. (2006). Effects of impurities containing phosphorus on the surface properties and catalytic activity of TiO_2_ nanotube arrays. Appl. Surf. Sci..

[13] Kwoka M., Galstyan V., Comini E., Szuber J. (2017). Pure and highly Nb-doped titanium dioxide nanotubular arrays: characterization of local surface properties. Nanomaterials.

[14] Iida Y., Ozaki S. (1961). Grain growth and phase transformation of titanium oxide during calcination. J. Am. Ceram. Soc..

[15] Shannon R.D., Pask J.A. (1965). Kinetics of the Anatase-Rutile Transformation. J. Am. Ceram. Soc..

[16] Low I.M., Albetran H., Prida V.M., Vega V., Manurung P., Ionescu M. (2013). A comparative study on crystallization behavior, phase stability, and binding energy in pure and Cr-doped TiO_2_ nanotubes. J. Mater. Res..

[17] Albetran H., O'Connor B., Low I. (2017). Effect of pressure on TiO_2_ crystallization kinetics using in-situ high-temperature synchrotron radiation diffraction. J. Am. Ceram. Soc..

[18] Liu R., Qiang L.S., Yang W.D., Liu H.Y. (2013). The effect of calcination conditions on the morphology, the architecture and the photo-electrical properties of TiO_2_ nanotube arrays. Mater. Res. Bul..

[19] Gamboa J.A., Pasquevich D.M. (1992). Effect of chlorine atmosphere on the anatase-rutile transformation. J. Am. Ceram. Soc..

[20] Li H., Cao L., Liu W., Su G., Dong B. (2012). Synthesis and investigation of TiO_2_ nanotube arrays prepared by anodization and their photocatalytic activity. Ceram. Int..

[21] Gong D., Grimes C.A., Varghese O.K., Hu W., Singh R.S., Chen Z., Dichey E.C. (2001). Titanium oxide nanotube arrays prepared by anodic oxidation. J. Mater. Res..

[22] Prida V.M., Hernández-Vélez M., Pirota K.R., Menéndez A., Vázquez M. (2696). Synthesis and magnetic properties of Ni nanocylinders in self-aligned and randomly disordered grown titania nanotubes. Nanotechnology.

[23] Tan A.W., Pingguan-Murphy B., Ahmad R., Akbar S.A. (2012). Review of titania nanotubes: Fabrication and cellular response. Ceram. Int..

[24] Arunchandran C., Ramya S., George R.P., Mudali U.K. (2013). Corrosion inhibitor storage and release property of TiO_2_ nanotube powder synthesized by rapid breakdown anodization method. Mater. Res. Bull..

[25] Liao J., Lin S., Pan N., Li D., Li S., Li J. (2012). Free-standing open-ended TiO_2_ nanotube membranes and their promising through-hole applications. Chem. Eng. J..

[26] Kim C.W., Suh S.P., Choi M.J., Kang Y.S., Kang Y.S. (2013). Fabrication of SrTiO_3_-TiO_2_ heterojunction photoanode with enlarged pore dimeter for dye-sensitized solar cells. J. Mater. Chem. A.

[27] Ding J., Huang Z., Zhu J., Kou S., Zhang X., Yang H. (2015). Low-temperature synthesis of high-ordered anatase TiO_2_ nanotube array films coated with exposed {001} nanofacets. Sci. Rep..

[28] Varghee O.K., Gong D.W., Paulose M., Grimes C.A., Dichey E.C. (20031). Crystallization and high-temperature structural stability of titanium oxide nanotubes arrays. J. Mater. Res..

[29] Yu J., Dai G., Cheng B. (2010). Effect of Crystallization Methods on Morphology and Photocatalytic Activity of Anodized TiO_2_ Nanotube Array Films. J. Phys. Chem. C.

[30] Albetran H., Low I.M. (2016). Effect of indium ion implantation on crystallization kinetics and phase transformation of anodized titania nanotubes using in-situ high-temperature radiation diffraction. J. Mater. Res..

[31] Albetran H., Haroosh H., Dong Y., Prida V.M., O’Connor B.H., Low I.M. (2014). Phase transformations and crystallization kinetics in electrospun TiO_2_ nanofibers in air and argon atmospheres. Appl. Phys. A.

[32] Albetran H., O'Connor B., Low I. (2016). Activation energies for phase transformations in electrospun titania nanofibers: comparing the influence of argon and air atmospheres. Appl. Phys. A.

[33] Cahn J.W. (1956). Transformation kinetics during continuous cooling. Acta. Metall..

[34] Galwey A.K., Brown M.E. (2002). Application of the Arrhenius equation to solid state kinetics: can this be justified?. Thermochim. Acta.

[35] Low I.M., Yam F., Pang W.K. (2012). In-situ diffraction studies on the crystallization and crystal growth in anodized TiO_2_ nanofibres. Mater. Lett..

[36] Albetran H., O'Connor B.H., Prida V.M., Low I.M. (2015). Effect of vanadium ion implantation on the crystallization kinetics and phase transformation of electrospun TiO_2_ nanofibers. Appl. Phys. A.

[37] Kumar K.N., Engell J. (1995). Pore-structure stabilization by controlling particle coordination. J. Mater. Sci. Lett..

[38] Kumar K.N.P., Keizer K., Burggraaf A.J. (1993). Textural stability of titania–alumina composite membranes. J. Mater. Chem..

